# Feasibility of Allogeneic Hematopoietic Stem Cell Transplantation Following Recent Invasive Mold Disease in Pediatric Patients

**DOI:** 10.3390/jof12040297

**Published:** 2026-04-21

**Authors:** Kevin Martínez Pérez, Daniela Torres-Hernández, Nathalia Sanclemente, Oscar Ramirez, Andrés Portilla, Jorge Buitrago, Eduardo López-Medina

**Affiliations:** 1 Department of Pediatrics, Universidad del Valle, Cali 760000, Colombia; ksmartinez19@gmail.com (K.M.P.); danitorres0890@gmail.com (D.T.-H.); 2 Clínica Imbanaco, Grupo Quirón Salud, Cali 760000, Colombia; natys0927@gmail.com (N.S.); oscar.ramirezw@quironsalud.com (O.R.); carlos.portilla@quironsalud.com (A.P.); jorge.buitrago@correounivalle.edu.co (J.B.); 3 Department of Scientific Advancement, Endpoints Network of Research Sites, Cali 760000, Colombia

**Keywords:** hematopoietic stem cell transplantation, invasive fungal disease, immunocompromised host, pediatric, Latin America

## Abstract

A history of invasive mold disease (IMD) often delays or contraindicates allogeneic hematopoietic stem cell transplantation (allo-HSCT) in children. Given the limited data on pediatric patients with pre-allo-HSCT IMD, we aimed to describe the management and clinical outcomes of a cohort of children with IMD prior to allo-HSCT through day +100 post-transplantation. Between 2021 and 2024, ten pediatric patients were identified with proven or probable IMD. Their median age was 8.5 years. The most common pathogens were *Aspergillus* (n = 5) and *Fusarium* (n = 4). Infections most frequently involved the lungs followed by paranasal sinuses, bloodstream, liver, and skin. All patients demonstrated clinical improvement before transplantation, and by day +100 post-HSCT, no IMD relapses or infection-related mortality were observed. These findings suggest that complete radiologic or clinical resolution is not a prerequisite for proceeding with transplantation. Recent IMD should not be considered an absolute contraindication to urgent allo-HSCT when clinical improvement is evident, as transplantation facilitates immune reconstitution necessary for definitive infection control.

## 1. Introduction

Invasive mold diseases (IMD) are associated with substantial morbidity and mortality among immunocompromised children and have historically been considered a relative contraindication to allogeneic hematopoietic stem cell transplantation (allo-HSCT) [1,2]. Concerns regarding infection reactivation or progression in the setting of post-transplant immunosuppression have often led to delays or deferral of transplantation [3,4].

Although advances in antifungal therapy, diagnostics, and transplant practices have improved outcomes, evidence guiding the management of children with pre-allo-HSCT IMD remains limited [3,5]. Most available data derive from adult cohorts, with little pediatric-specific guidance on the optimal timing of allo-HSCT following IFI or on the clinical, radiologic, and microbiologic criteria used to assess transplant readiness [3,5].

As the number of children undergoing allo-HSCT continues to increase, cumulative exposure to risk factors for IMD prior to transplantation is also rising [6]. Data from low- and middle-income countries (LMICs) are particularly scarce, despite the high burden of infectious complications in these settings. In this context, we sought to evaluate the impact of prior IMD on post–allo-HSCT mortality in children and to assess whether, and under what circumstances, allo-HSCT can be performed following IMD in this vulnerable population.

## 2. Materials and Methods

### 2.1. Study Design and Population

We conducted a retrospective cohort study of all pediatric patients with IMD prior to allo-HSCT at Clínica Imbanaco, a tertiary referral center in Cali, Colombia, between January 2021 and January 2024. The study was approved by the institutional ethics committee.

Patients were eligible if they were <18 years of age, had a proven or probable IMD within 120 days before allo-HSCT, and had clinical follow-up for at least 100 days after transplantation.

### 2.2. Data Sources and Definitions

Patients were prospectively identified through the institutional allo-HSCT infectious diseases registry. Retrospective data were abstracted from electronic medical records using a standardized data collection approach.

IMD was defined according to the European Organization for Research and Treatment of Cancer/Mycoses Study Group (EORTC/MSG) consensus criteria [7]. Diagnosis, antifungal management, and longitudinal assessment were performed by the infectious diseases team of the allo-HSCT unit in accordance with institutional protocols based on international guidelines [8,9,10,11,12,13].

### 2.3. Outcomes

Medical records were reviewed through day 100 post–allo-HSCT to ascertain outcomes, including IMD relapse and IMD-related mortality, defined as death primarily attributable to IMD as documented by the treating clinical teams.

### 2.4. Transplant Procedures

Conditioning regimens were administered according to standard institutional protocols. For malignant indications, myeloablative conditioning regimens included fludarabine with total body irradiation; fludarabine, busulfan, and melphalan; or fludarabine with etoposide. Patients with bone marrow failure syndromes received fludarabine and cyclophosphamide combined with thymoglobulin and either total body irradiation or melphalan. One patient with an inborn error of immunity did not receive conditioning.

Graft-versus-host disease (GVHD) prophylaxis included post-transplant cyclophosphamide, abatacept, and cyclosporine, according to institutional practice.

### 2.5. Statistical Analysis

Descriptive statistics were used to summarize patient characteristics and outcomes. Continuous variables are reported as medians with interquartile ranges (IQRs), and categorical variables as frequencies and percentages. Given the descriptive nature and sample size of the cohort, no comparative analyses were performed. All statistical analyses were conducted using Stata software, version 18.0 (StataCorp, College Station, TX, USA).

## 3. Results

During the study period (January 2021–December 2024), 133 pediatric hematopoietic stem cell transplants were performed, including 108 allogeneic procedures. Ten patients requiring allo-HSCT had a history of proven or probable IFI within the preceding 120 days and were included in the analysis.

Their median age was 8.5 years (range, 3 months–16 years), and five were male. Primary indications for allo-HSCT were acute lymphoblastic leukemia (n = 4) and acute myeloid leukemia (n = 4). IMD was diagnosed a median of 45 days before allo-HSCT (IQR, 21–106) (Table 1). At the time of IFI diagnosis, nine patients were receiving antifungal prophylaxis, most commonly fluconazole (n = 4) or voriconazole (n = 4). Voriconazole trough concentrations were not assessed during prophylaxis. The median duration of neutropenia prior to IFI diagnosis was 22 days (IQR, 14 to 26.5).

Common presenting features included fever (n = 9), cough (n = 5), and sinonasal symptoms with maxillary or frontal pain (n = 3). One afebrile patient presented with respiratory distress. Three patients required intensive care unit admission prior to HSCT due to IMD. The most frequently identified pathogens were *Aspergillus* spp. (n = 5) and *Fusarium* spp. (n = 4); *Candida*, *Mucor*, and *Trichosporon* species were each identified in one patient (Table 1).

The lung was the most commonly involved site (n = 9), followed by paranasal sinuses, bloodstream, liver, and skin (n = 3 each), heart (n = 2), and kidney and retina (n = 1 each). Pulmonary imaging most frequently demonstrated nodules (n = 6) and ground-glass opacities (n = 5). Other findings included interstitial or reticular infiltrates, pleural effusions, and halo sign (n = 2 each), as well as consolidation, bronchiectasis, and atelectasis (n = 1 each) (Table 1).

Combination antifungal therapy was administered to seven patients, most commonly amphotericin B plus voriconazole (n = 4) (Table 2). Amphotericin B was administered at a mean dose of 5.1 mg/kg/day, and voriconazole at 20.7 mg/kg/day. Among the eight patients who received voriconazole as definitive therapy, serum drug concentrations were measured in all, with a mean trough level of 2.31 µg/mL (SD, 1.25).

Five patients required surgical intervention for IMD a median of 31.5 days (IQR 14–49) prior to allo-HSCT, and two patients underwent surgical intervention for IFI after HSCT at a mean of 71 days.

Allo-HSCT was performed a median of 45 days after IMD diagnosis (IQR, 21–106). All patients demonstrated clinical improvement prior to initiation of conditioning (Table 2). Fever resolved a median of 24 days before allo-HSCT (IQR, 13 to 58), following a median duration of 11 days after IMD diagnosis (IQR, 6 to 23). Improvement in inflammatory markers, fever resolution, and radiologic findings was observed in 9, 8 and 7 of patients, respectively (Table 2).

One patient with an urgent indication for transplantation, initiated conditioning 12 days after IMD diagnosis, at which time clinical improvement had been documented despite persistent fever and worsening radiologic findings.

Through day 100 post-transplant, no patient experienced IMD relapse; all patients demonstrated continued clinical and radiologic improvement, and no IMD-related mortality was observed.

## 4. Discussion

In this cohort of pediatric patients who developed invasive mold disease prior to allogeneic hematopoietic stem cell transplantation, all ultimately proceeded to transplantation and demonstrated clinical and radiologic improvement following allo-HSCT. Notably, no patient experienced IFI-related mortality or post-transplant relapse of fungal infection.

Considering the well-established risk factors for IMD-related complications in the post-transplant period—including cytomegalovirus infection, residual disease, corticosteroid exposure, severe GVHD, and prolonged neutropenia [14,15,16], many of which are inherent to conditioning regimens—earlier studies reported high rates of post-transplant morbidity and mortality in patients with pre-transplant IMD [17,18,19]. Accordingly, pre-transplant IMD was historically considered a relative or absolute contraindication to allo-HSCT [3,5,20]. However, more recent data suggest that improved outcomes are achievable, likely reflecting more timely initiation of antifungal therapy and the availability of better and newer antifungal agents [21,22,23,24,25,26,27,28]. Advances in diagnostic modalities facilitate earlier recognition of fungal activity, and are useful diagnostic and prognostic tools; nevertheless, suboptimal diagnostic accuracy remains a common limitation [29]. In this cohort, *Aspergillus* galactomannan likely cross-reacted with the galactofuranose-containing antigens of *Fusarium* [30,31], resulting in a false-positive finding, and was falsely negative in a proven case of proven *Aspergillus* pneumonia. These observations underscore the need for more accurate diagnostic modalities in pediatric patients.

Although current guidelines do not routinely recommend antifungal combination therapy, most patients in this series received voriconazole in combination with liposomal amphotericin B, reflecting perceived disease severity and the urgency of infection control prior to transplantation. Importantly, three patients were successfully managed with voriconazole monotherapy, suggesting that combination therapy may not be universally required, even in complex pre-transplant settings. These findings underscore the need for individualized therapeutic decision-making based on clinical trajectory and host factors.

Management strategies across this cohort were consistent with published IMD treatment guidelines [9,10,11,12,13], emphasizing prompt antifungal therapy, surgical source control when feasible, therapeutic drug monitoring, reduction in immunosuppression when possible, and intensive supportive care. Despite these measures, determining the optimal timing of allo-HSCT in the setting of recent IMD remains challenging. While guidelines generally recommend a minimum of six to eight weeks of antifungal therapy prior to transplantation [3,29,32], many patients require urgent HSCT because of the high risk of progression of the underlying disease. Moreover, immune reconstitution—particularly neutrophil recovery—represents a critical determinant of IMD control, thereby providing a rationale for proceeding with transplantation, according to procedural urgency, once clinical improvement is evident [20,30,33].

Most patients initiated conditioning chemotherapy for allo-HSCT after demonstrating clinical, radiologic, and inflammatory improvement. However, one patient with an urgent indication for transplantation received donor cell infusion 12 days after IFI diagnosis despite persistent fever and worsening radiographic findings. In addition, one patient exhibited radiologic progression, and another remained febrile despite appropriate antifungal therapy and surgical source control. All three patients had persistent neutropenia that was unlikely to resolve without allo-HSCT and demonstrated overall clinical improvement prior to proceeding with transplantation.

Complete eradication of IFI prior to transplantation is often difficult to achieve, particularly in patients with profound or prolonged neutropenia or in those requiring urgent HSCT. Although the risk of infectious complications remains elevated in this setting, our findings provide proof of concept that allo-HSCT is feasible and can be pursued once meaningful clinical improvement in IFI manifestations is observed. Complete clinical or radiologic resolution may not be rapidly attainable and does not appear to be essential. Accordingly, a recent IMD should not be considered an absolute contraindication to urgent allo-HSCT when careful multidisciplinary management and close monitoring are in place.

## Figures and Tables

**Table 1:** Baseline Demographic and Clinical Characteristics of Pediatric Patients With Invasive Mold Disease Prior to Allogeneic Hematopoietic Stem Cell Transplantation.

Case	Age	Sex	Race	Indication for Allo-HSCT	Duration of Neutropenia in Days Before IMD	Signs and Symptoms of IMD	Duration of Signs and Symptoms in Days at the Time of IMD Diagnosis	Involved Organs	Etiologic Agent/ EORTC
1	8 Y	F	Mixed	Bone marrow failure syndrome	14	Cough, fever, nasal congestion, facial pain	14	Lungs and paranasal sinuses	*Fusarium* spp./Proven
2	7 Y	F	Mixed	ALL	14	Cough, fever, nasal congestion	12	Lungs, liver and heart, blood	*Aspergillus* spp./Probable & *Candida parapsilosis*/Proven
3	15 Y	M	Mixed	AML	27	Fever	13	Blood, skin, heart, paranasal sinuses	*Fusarium solani*/Proven
4	3 M	M	Indigenous	Inborn error of immunity	9	Respiratory distress	2	Lungs	*Aspergillus* spp./Probable
5	9 Y	M	Mixed	ALL	7	Fever	10	Lungs	*Aspergillus* spp./Probable
6	10 Y	M	Mixed	AML	25	Fever and diarrhea	5	Lungs and liver	*Mucor* spp./Proven
7	8 Y	F	Mixed	ALL	35	Cough, fever	7	Right hand joints, paranasal sinuses and lungs	*Fusarium* spp./Proven
8	16 Y	F	Indigenous	AML	82	Fever	3	Lungs	*Aspergillus* spp./Proven
9	11 Y	M	Indigenous	ALL	22	Cough, fever	6	Blood, skin, retina, lungs, liver and kidneys	*Trichosporon asahii*/Proven & *Fusarium* spp./Proven
10	11 Y	F	Mixed	AML	22	Cough, fever, nasal congestion, and facial pain	5	Lungs and paranasal sinuses	*Aspergillus Flavus*/Proven

Y, years; M, months; F, female; M, male; ALL, acute lymphoblastic leukemia; AML, acute myeloid leukemia; IMD, invasive mold disease.

**Table 2:** Clinical Characteristics, Antifungal Management, and Infection Status of Patients With Invasive Mold Disease Prior to Allogeneic Hematopoietic Stem Cell Transplantation.

Case	Duration of Neutropenia in Days After IMD Diagnosis	Duration of Fever in Days After IMD Dx	ICU Requirement Due to IMD Prior to Allo-HSCT	Surgical Control/Days of Allo-HSCT	Backbone Antifungal Therapy for IMD	Days of Antifungal Therapy Between IMD and Allo-HSCT	Imaging Results before Allo-HSCT	IFI-Related Clinical Condition at Time of Allo-HSCT	GM at Dx and at the Time of Allo-HSCT	PCT at Dx and at the Time of Allo-HSCT
**1**	10	37	No	Yes/−49	Vori & L-AmB	50	Improv.	Improv., became afebrile	0.31/ 0.18	83.06/0.29
**2**	13	10	No	No	Vori & Anidula	45	Improv.	Improv., became afebrile	2.86/ 0.56	76.24/0.12
**3**	12	12	No	Yes/ +64, +99	Vori & L-AmB	12	Det.	Improv. but remained febrile	0.18/-	0.05/ 0.59
**4**	2	1	No	No	Vori	28	Improv.	Improv., became afebrile	0.34/-	0.38/ 0,08
**5**	98	1	Yes	No	Vori	118	Improv.	Improv., became afebrile	0.64/ 0.37	3.32/ 0.06
**6**	45	24	No	Yes/+49	Posa & L-AmB	45	Det.	Improv., became afebrile	-	6.28/ 1.46
**7**	21	19	No	Yes/−82	Vori & L-AmB	106	Improv.	Improv., became afebrile	10.1/0.35	0.81/ 0.18
**8**	21	5	No	Yes/−14	Vori & caspo	21	No change	Improv., became afebrile	3.13/ 1.23	0.14/ 0.07
**9**	6	49	Yes	Yes/ −49, −42, −35, −28	Vori & L-AmB	107	Improv.	Improv. but remained febrile	0.26/ 0.22	2.16/ 0.09
**10**	14	10	Yes	Yes/ −6	Vori	14	Improv.	Improv., became afebrile	-/2.85	1.21/ 0.83

Vori, voriconazole; L-AmB, liposomal amphotericin B; anidula, anidulafungin; posa, posaconazole; caspo, caspofungin; improv., improvement; det., deterioration; GM, galactomannan; dx, diagnosis; PCT, procalcitonin.

## Data Availability

The original contributions presented in the study are included in the article, further inquiries can be directed to the corresponding authors.

## Abbreviations

The following abbreviations are used in this manuscript:

IMD	Invasive mold diseases
IFI	invasive filamentous fungal infection
allo-HSCT	allogeneic hematopoietic stem cell transplantation
LMICs	low- and middle-income countries
GVHD	Graft-versus-host disease
IQRs	interquartile ranges
